# Analysis of two cases with bronchopulmonary neurofibromatosis

**DOI:** 10.1186/2049-6958-7-17

**Published:** 2012-07-18

**Authors:** Ting Yuan, Bai-ling Luo, Qi-hua Gu, Ji Yao

**Affiliations:** 1 Department of Respiratory Medicine, Xiang Ya Hospital, Central South University, Changsha, Hunan province 410008, China; 2 Department of Radiological Medicine, Xiang Ya Hospital, Central South University, Changsha, Hunan province 410008, China

**Keywords:** Endobronchial, Neurofibromatosis, Pleural

## Abstract

Neurogenic tumor of lung is very rare. Only few cases have been reported in the literature. We present here two cases of bronchopulmonary neurofibromatosis in two adults. In both cases, attempts at imaging failed to diagnose the case, and it was the histological study that ensured the diagnosis of neurofibromatosis. Biopsy specimens showed bundles of spindle-shaped cells mixed with collagen, and on immunohistochemistry some cells were positive for S-100 protein.

## Cases

### Case 1

**Figure 1:**
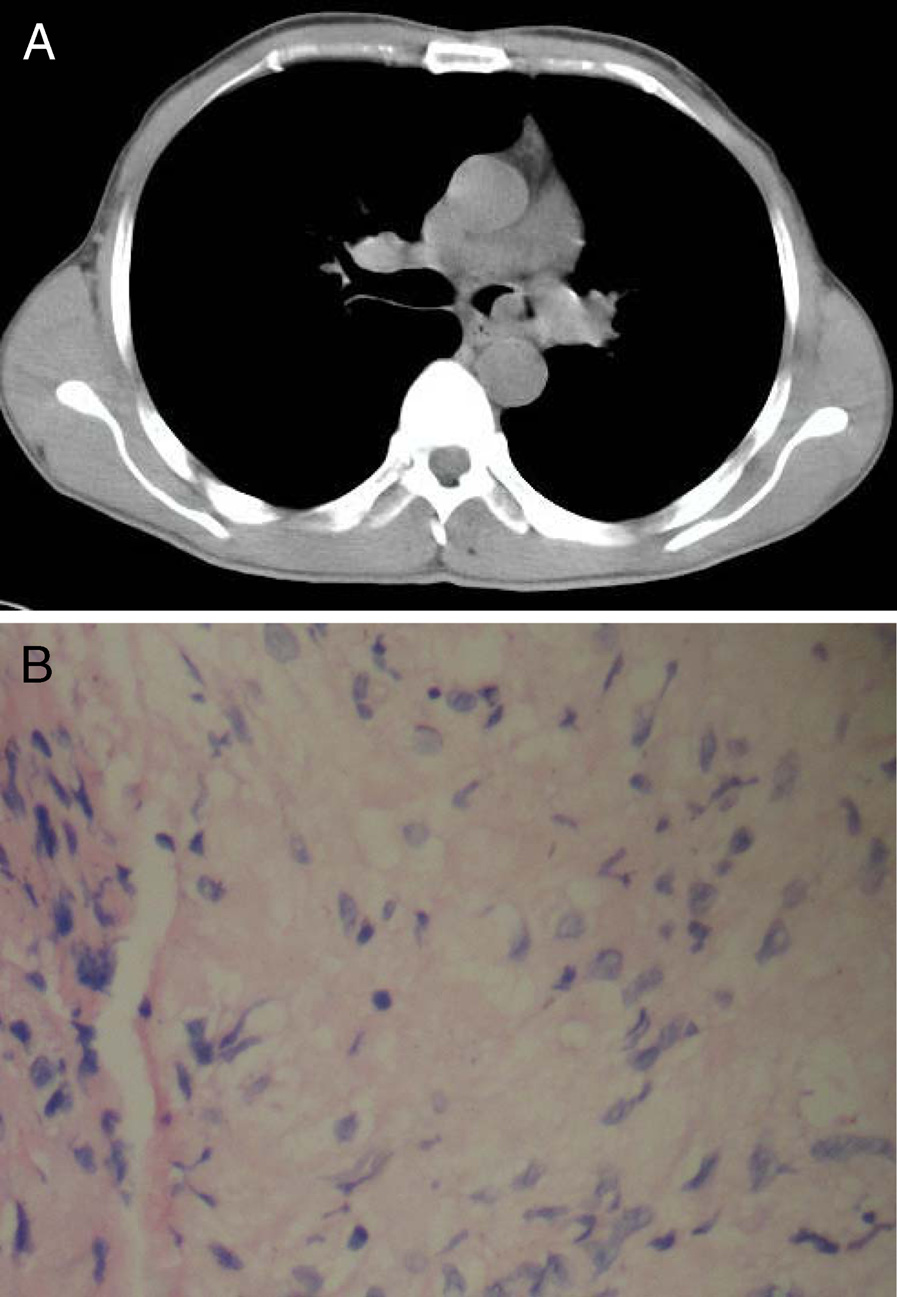
**A - Chest CT showing a nodule in left bronchus. B -** Pathological section showing interlacing bundles of spindle-shaped cells mixed with collagen. HE × 400.

A 47-year-old male was admitted to the Respiratory Medicine department of Xiangya Hospital on March 29^th^, 2010 complaining of repeated cough and expectoration for 5 years, aggravated with intermittent fever for 4 months. From 2005, the patient had begun to have cough and expectoration after a common cold, and this symptom became more serious in December 2009 when intermittent fever occurred. His body temperature became more than 39°C and he got dyspnea when the temperature was abnormal. The outpatient chest X-ray showed infection of the lower left lung and a small quantity of pleural effusion. Relief of symptoms of fever and cough was obtained after treatment with cephalosporin, but the symptoms would recur 1 or 2 weeks later. In order to get better treatment he came to our department. He had no hypertension or diabetes, and had a long history of smoking. Physical examination on admission showed no abnormal findings on the skin, and the lymph nodes were impalpable. The left lung was dull at percussion. Breath sound of the left lung was coarse and moist rales could be heard. Examination of the heart and abdomen were normal. The laboratory test, complete blood count (CBC), white blood cells (WBC) 29.8 x 10^9^/L, neutrophils 87%, sputum culture, HIV-antibody and carcinoembryonic antigen (CEA) were all normal. Chest computed tomography (CT) revealed a nodule in the left bronchus, obstructive pneumonia in the lower left lung and lymphadenectasis in the mediastinum (Figure 1A). The primary diagnosis was tumor in the left bronchus with obstructive pneumonia in the left lower lung. He was given antibiotics treatment. Bronchoscopy showed a spherical tumor with amicula in the bronchus cavity, which was located in the lateral wall 5 mm far from the eminence. The tumor was smooth and rich in blood vessels. The cavity was almost obstructed. Excision biopsy and histology of the nodule showed encapsulated tumor tissue composed of interlacing bundles of spindle-shaped cells mixed with collagen (Figure 1B). On immunohistochemistry some cells were positive for S-100 protein and CD-56. The final diagnosis was left bronchus neurofibromatosis, and the patient subsequently underwent tumor resection in the Thoracic department.

### Case 2

**Figure 2:**
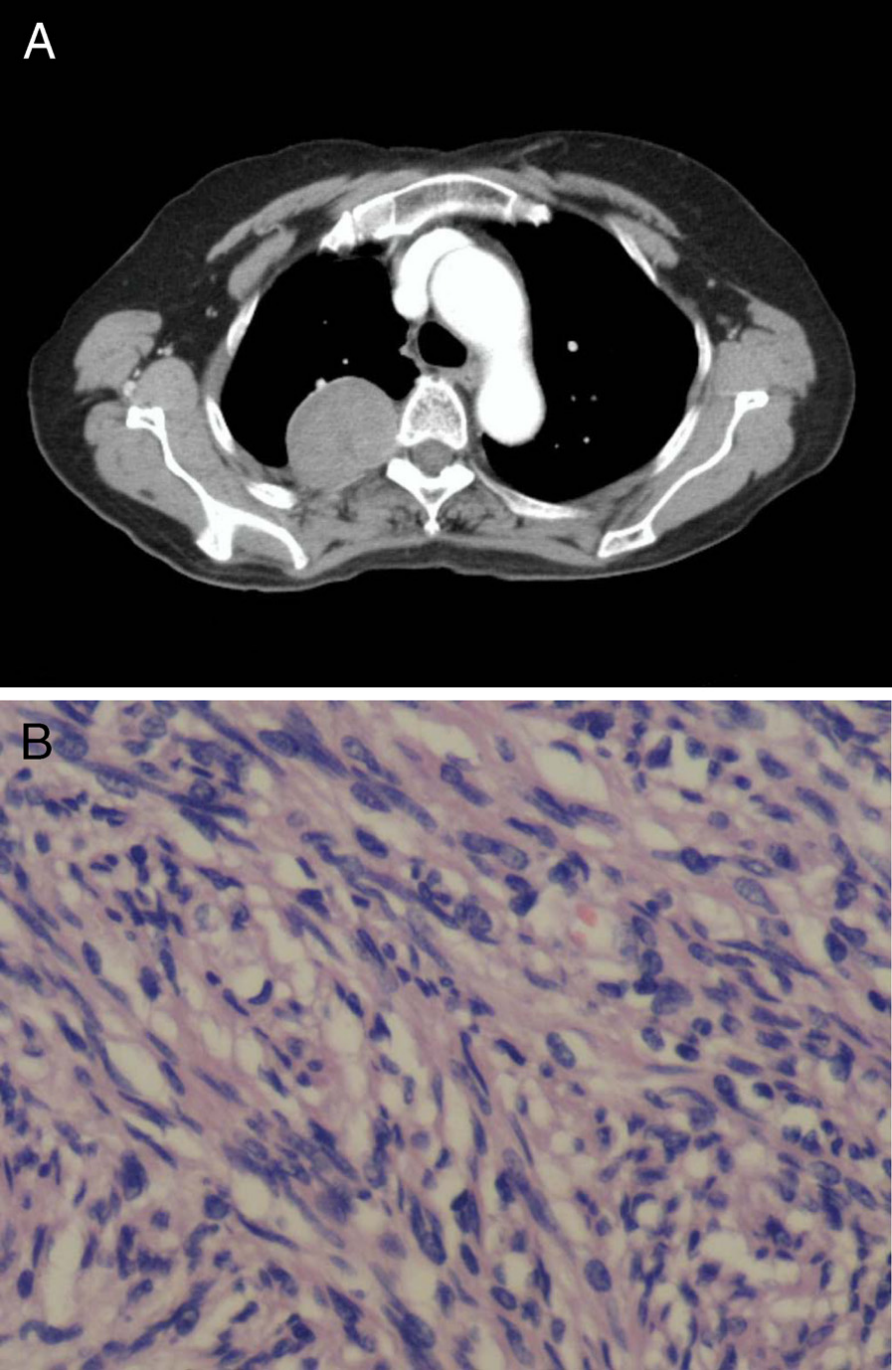
**A - Chest CT scan showing a soft tissue density in the right upper lung. B -** Pathological section showing bundles of spindle-shaped cells. HE × 400.

A 66-year-old female was admitted to the Respiratory Medicine department of Xiangya Hospital on April 12^th^, 2010 complaining of cough and expectoration for 1 year, and breathlessness and bloody sputum for half a year, aggravated for 1 month. From March 2009, the patient had begun to experience cough and expectoration, but did not receive any treatment. Her sputum began to be mixed with cherry blood streak in October 2009 and in the course of one month her symptoms became more serious. The local hospital gave her anti-inflammatory therapy, and the symptoms of cough were relieved. But bloody sputum persisted, and breathlessness on exertion became more serious. The local chest CT showed a tumor in the right upper lung. In order to get better treatment she came to our department. The past and family histories were unremarkable, and the patient did not have a history of smoking. Physical examination on admission showed no abnormality. Chest examination showed no abnormal findings. There were no café-au-lait spots, frecklings in the axilla or groin, or cutaneous nodules. CBC, the sputum culture, HIV-antibody, and CEA were all normal. Chest CT showed a lesion in the right upper chest wall and right pleural effusion (Figure 2A). Bronchoscopy failed to evidence any obvious abnormality. CT-guided percutaneous transthoracic aspiration biopsy of lung lesions showed bundles of spindle-shaped cells mixed with collagen (Figure 2B). On immunohistochemistry some cells were positive for S-100 protein. The final diagnosis was right pleural neurofibromatosis and she underwent resection. During the operation, a tumor of about 3 x 3 cm could be seen located on the partial dorsal pleura, it had complete amicula, and the pedicle initiated from intercostal nerve.

## Discussion

Neoplasias today represent the second main cause of death throughout the world, and tumors of the respiratory system occupy first position as a cause of mortality both as regards males and females [1]. Among these, chest primary neurogenic tumors have predilection in the back mediastina and costovertebral angle, but tumors arising from the tracheobronchial tree or lung are very rare. Neurofibromatosis is a benign tumor which consists of neurilemma cells and fibroblasts. It can develop anywhere on the peripheral nerve, and can be seen most frequently on the trunk, lower extremity, head, and upper extremity (in order of occurrence), but less in the mediastinum, perineum and so on; however, respiratory neurofibromatosis is very uncommon [2]. In the case of lung involvement it can cause obstruction, chest deformities, airway and parenchymal neurogenic tumors, pulmonary fibrosis, cystic pulmonary diseases, primary pulmonary hypertension, central hypoventilation and diaphragm paralysis and so on [3]. The initial symptoms include cough, wheezing, and dyspnea, which are related to airway obstruction. However the majority of patients have normal chest x-ray on initial presentation [4]. Patients with tracheobronchial tumors may be asymptomatic for years. Chest roentgenography may reveal infiltration and atelectasis, though the majority of patients have normal chest films on initial presentation. Delayed diagnosis is not unusual, as overlying soft tissue obscures the trachea in posteroanterior radiography [5]. Surgical resection of the lesions can often be technically difficult, and sometimes surgical resection or biopsy can result in motor or sensory loss. There is also the possibility that surgical trauma can act as a stimulus for recurrence and malignant transformation [6].

The first patient’s main manifestations were repeated cough and expectoration, which were relieved after anti-inflammatory treatment; so the clinic physician only paid attention to his pneumonia, but never deeply analyzed the reason why he got pneumonia repeatedly. But the patient had repeated pneumonia, and the chest X-ray could be normal after anti-inflammatory treatment. This is the reason why the real etiology was misdiagnosed for 5 years. If he had been diagnosed when the tumor was small, it would have been possible to resect the tumor under the bronchoscope, e.g. by transbronchial electrical snaring and Nd-YAG laser abrasion [5], which would have avoided tissue damage caused by open thoracotomy and reduced the patient’s financial burden.

Pleural neurofibromatosis initiates from the intercostal nerve, manifesting as chest distress, and dyspnea on exertion. Langman and coll. [7] reported two cases of pleural neurofibroma, who also had shoulder pain, and did not have any chest related symptoms. Our second patient’s symptoms were cough, expectoration, bloody sputum and dyspnea on exertion. The dyspnea on exertion may have been due to pleural effusion. The patient was not given a chest CT until the bloody sputum occurred. Chest CT showed a lesion in the right lung and right pleural effusion. Lung cancer or the pleural tumor were first considered. Pleural neurofibromas are characterised by wavy elongated cells and associated strands of collagen, separated by mucoid material. Histologically they are characterised by spindled, wavy dispersed cells separated by a collagenous and myxoidstroma. Mast cells are frequently present and are a helpful diagnostic feature. In our cases, CT-guided percutaneous transthoracic aspiration biopsy of lung showed bundles of spindle-shaped cells mixed with collagen. On immunohistochemistry some cells were positive for S-100 protein, proving the presence of pleural neurofibroma.

## Conclusions

The above two cases showed that all patients affected by pneumonia - especially a local repeated pneumonia - should undergo essential auxiliary examinations. Chest lamellar CT is helpful to find small lesions in airway or lesions in the lung; bronchoscopy is not only used to explore the airway, but also to biopsy in order to understand the etiology. After diagnosis of the neurofibromas, the treatment depends on the size and the location of the tumors, and should be conservative. Some small tumors can be removed endoscopically with or without laser. However, if the tumors invade adjacent tissue, surgery should be carried out.

## Consent

Written informed consent was obtained from the patient for publication of this case report and any accompanying images. A copy of the written consent is available for review by the Editor-in-Chief of this journal.

## Competing interests

The authors declare that they have no competing interests.
